# Mortality following emergency laparotomy: a Swedish cohort study

**DOI:** 10.1186/s12893-021-01319-8

**Published:** 2021-08-11

**Authors:** Terje Jansson Timan, Gustav Hagberg, Ninni Sernert, Ove Karlsson, Mattias Prytz

**Affiliations:** 1 Department of Surgery, Institute of Clinical Sciences, Sahlgrenska Academy, University of Gothenburg, Gothenburg, Sweden; 2 Department of Research and Development, NU-Hospital Group, Trollhättan, Sweden; 3 Department of Anesthesiology and Intensive Care Unit, NU-Hospital Group, Trollhättan, Sweden; 4 Department of Surgery, NU-Hospital Group, Trollhättan, Sweden; 5 Department of Orthopedics, Institute of Clinical Sciences, Sahlgrenska Academy, University of Gothenburg, Gothenburg, Sweden; 6 Department of Anesthesiology and Intensive Care, Institute of Clinical Sciences, Sahlgrenska Academy, University of Gothenburg, Gothenburg, Sweden

**Keywords:** Acute surgery, Emergency laparotomy, Acute abdomen, Outcome, Mortality, Intensive care, ICU, Perioperative management

## Abstract

**Background:**

Emergency laparotomy (EL) is a central, high-risk procedure in emergency surgery. Patients in need of an EL present an acute pathology in the abdomen that must be operated on in order to save their lives. Usually, the underlying condition produces an affected physiology. The perioperative management of this critically ill patient group in need of high-risk surgery and anaesthesia is challenging and related to high mortality worldwide. However, outcomes in Sweden have yet to be studied. This retrospective cohort study explores the perioperative management and outcome after 710 ELs by investigating mortality, overall length of stay (LOS) in hospital, need for care at the intensive care unit (ICU), surgical complications and a general review of perioperative management.

**Methods:**

Medical records after laparotomy was retrospectively analysed for a period of 38 months (2014–2017), the emergency cases were included. Children (< 18 years), aortic surgery, second look and other expected reoperations were excluded. Demographic, management and outcome data were collected after an extensive analysis of the cohort.

**Results:**

A total of 710 consecutive operations, representing 663 patients, were included in the cohort (mean age 65.6 years). Mortality (30 days/1 year) after all operations was 14.2% and 26.6% respectively. The mean LOS in hospital was 12 days, while LOS in the ICU was five days. Of all operations, 23.8% patients were admitted at any time to the ICU postoperatively and the 30-day mortality seen among ICU patients was 37.9%. Mortality was strongly correlated to existing comorbidity, high ASA classification, ICU care and faecal peritonitis. The mean/median time from notification to operate until the first incision was 3:46/3:02 h and 87% of patients had their first incision within 6 h of notification.

**Conclusions:**

In this present Swedish study, high mortality and morbidity were observed after emergency laparotomy, which is in agreement with other recent studies.

*Trial registration:* The study has been registered with ClinicalTrials.gov (NCT03549624, registered 8 June 2018).

## Background

General surgical procedures account for a large proportion of the care provided in hospitals in many countries [1]. Compared with emergency surgery, patients undergoing elective surgical procedures have a much lower mortality and morbidity rate. Elective surgery constitutes the vast majority of general surgical procedures [2, 3]. Acute abdominal surgery can be stratified by risk (high and low risk), where high-risk procedures require a significant amount of hospital resources and are associated with high mortality and morbidity [4, 5]. Emergency laparotomy (EL), a high-risk procedure, is a cornerstone of emergency surgery and is often performed when the clinically impaired patient requires urgent surgery for an acute abdominal condition. Although the underlying pathology varies, patients undergoing EL can be seen as a subgroup in the field with high mortality, especially among the elderly with comorbidity [6, 7]. International studies report a 7–21% short-term mortality rate, a long overall stay in hospital and a large number of ICU admissions for patients undergoing EL [6, 8–11].

Although patient demographics vary, a typical case may generally be an elderly patient with comorbidities and a pathological abdominal condition affecting organ function and generating critical illness, where the necessary action is an EL. It is a challenge for healthcare providers to handle these complex cases to ensure the best possible outcome [12]. In recent years, great efforts have been made to improve the postoperative outcome for patients undergoing an EL [11, 13, 14]. These studies indicate that the postoperative patient outcome can be improved by standardised perioperative management, a care bundle, with several key actions and measures, together with a high level of competence in the responsible surgeon and anaesthesiologist.

To the best of our knowledge, outcome after EL has not recently been studied in large Swedish cohorts. In this cohort study, we aim to explore the outcome after EL in 710 consecutive patients with respect to primary (30-day mortality) and secondary endpoints (overall hospital stay, number of ICU admissions/days in the ICU and surgical complications). In addition, the study also examines the surgical and anaesthesiological perioperative management of the cohort. During the current study period, no standardisation of perioperative management was carried out.

## Methods

### Study design

**Fig. 1:**
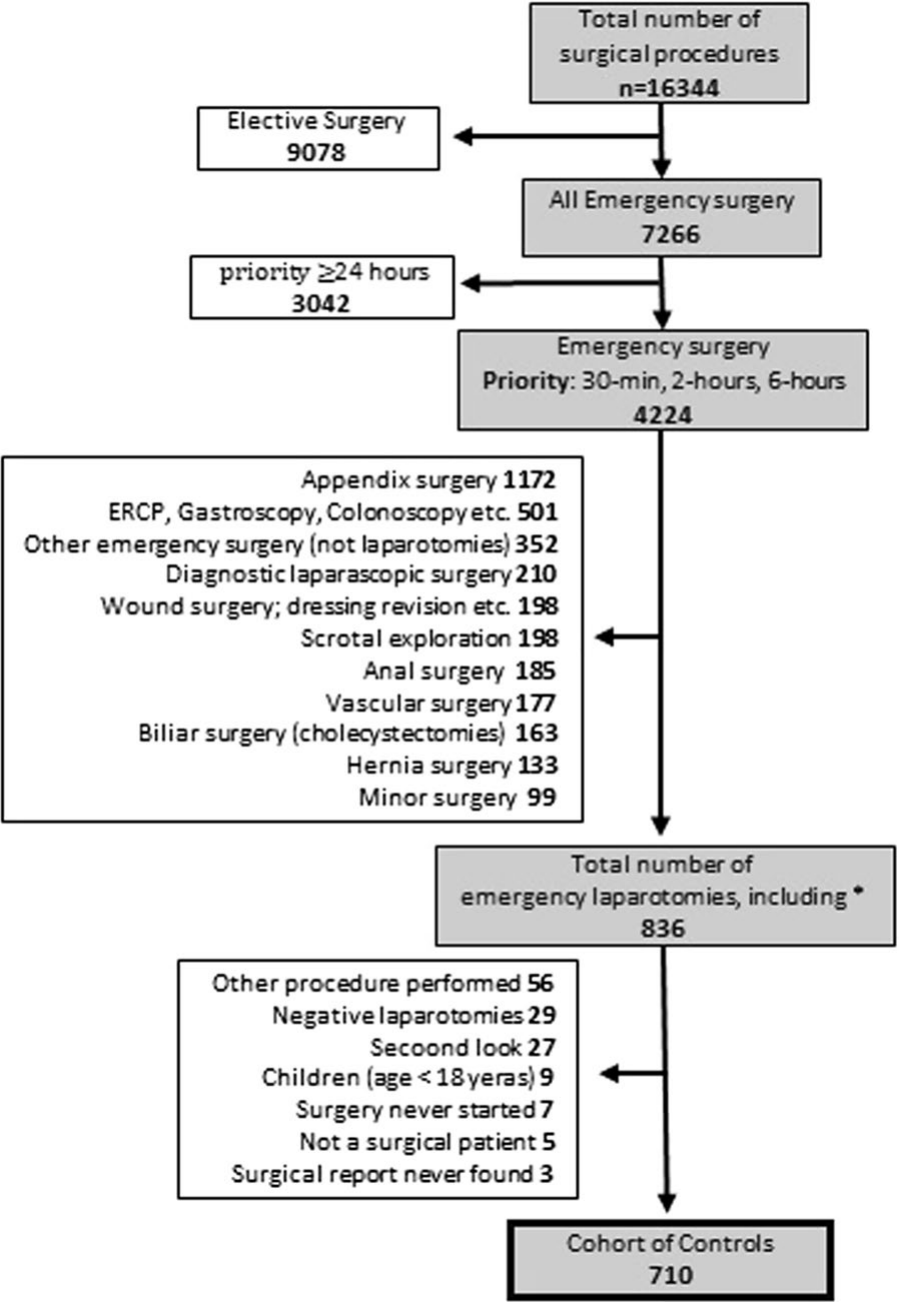
Study structure. This figure show the exclusion/inclusion process. The total number of surgical procedures performed during the study period of 38 months (2014–2017). *The laparotomies that had a different procedure-code initially

This single-centre retrospective cohort study aims to explore the outcome after EL in 710 consecutive operations (Fig. 1). Data on perioperative management and the demographic structure of the cohort were collected.

During the study period (20 August 2014 to 20 October 2017), there was no standardisation of perioperative management for these patients at our institution, NU-Hospital Group (NÄL; Trollhättan, Sweden). Perioperative management was at the discretion of the responsible surgeon and anaesthesiologist. Important management factors, such as the start of i.v. antibiotics, perioperative monitoring and postoperative assessment by the anaesthesiologist, could therefore vary. Even critical factors, like the time to surgery and perioperative interventions, differed between patients.

### Patient selection

The study period for this control cohort study was chosen (Fig. 1) because the standardisation of EL at NÄL started in February 2018. The current study aims to investigate the outcome before standardisation. The medical records for all cases undergoing EL during the study period were scrutinised. All adult patients (≥ 18 years) with a priority of six hours or less to the start of surgery were included. The priority for surgery was according to the discretion of the surgeon responsible for the patient and based on the patient’s clinical status and suspected underlying pathology. There was at the time no written standardisation for this decision in the department. Exclusions comprised acute aortic surgery, children (0–17 years), negative laparotomies (i.e. when no intra-abdominal pathology or injury was found), planned reoperations (for example, second-look surgery) and expected reoperations shortly after EL (when the patient does not improve clinically postoperatively).

### Demographic variables

To assess comorbidity, medical records were scrutinised for smoking, obesity, diabetes, renal failure, heart failure, ischaemic heart disease (IHD) and COPD (Chronic Obstructive Pulmonary Disease). In addition, data on current cancer diagnoses were collected and these data represent patients who have a registered cancer diagnosis for at least one hospital admission from three years before until one year after the date of surgery. For patients who underwent multiple operations, the time span is from three years before the first operation until one year after the last.

Pathological findings, such as mechanical ileus, peritonitis, ischaemic or gangrenous bowel, bleeding, perforation or trauma, supplemented the information. There were two possible ways of tracking the data. Firstly, the medical records where the prevalence of current diseases and treatments was stated. Secondly, the anaesthesiologist’s preoperative assessment from the surgical record, which stated current diseases, medications and results in an American Society of Anaesthesiologists (ASA) classification [15, 16]. An ASA classification was missing for 25 cases and a senior consultant at the Department of Anaesthesiology classified the missing cases retrospectively. Besides comorbidity, data for age, gender, height and weight were collected.

### Management variables

To illustrate the perioperative management, three types of variable were required: time-point variables, management variables and competence information.

Analysis of important time points: in the present study, the start of patient management was defined as the time of notification to operate. The time from the start until the first incision was analysed for the three priority categories, i.e. start within 30 min, 2 h or 6 h. The exact time points for the start of anaesthesia, the start of surgical operation (first incision), the end of operation, the start and end of care at the postoperative recovery unit, as well as the start and end dates for ICU care and overall hospital stay, were defined.

To assess the overall management performed, the surgical report and the computerised operational planning system (Orbit^©^) was used. The surgical procedures, such as adhesiolysis, intestinal resections, stoma, anastomoses and the removal of other organs, were summarised. The anaesthesiological assessment and interventions included data on the induction drug, treatment with vasopressors, arterial line insertion, application of epidural anaesthesia and treatment with perioperative antibiotics.

The level of competence in the operating theatre was divided into the three categories of registrar, specialist and senior consultant.

### Outcome variables

The main outcome (i.e. short-term mortality) was defined as mortality within 30 days from the first incision. The secondary outcomes included long-term mortality (within 90 and 365 days), overall LOS in hospital and the ICU and the need for intensive care. The Clavien–Dindo (C–D) classification system was used to identify and classify surgical complications. The cohort was divided into two groups representing less severe (C–D score of 1 to 3a) and major (C–D score of 3b to 5) complications [17]. The rationale behind this grouping is that C-D 1-3a are complications that require drug treatment, blood products or diagnostic procedures. The group with major complications has either undergone repeated surgery and intensive care or expired.

### Data and statistical analysis

In the inclusion phase, each patient's medical record data were scrutinised. Then, in the processing phase, all the data were analysed and de-identified. All the variable data were presented as frequency and percentage (n, %). Continuous variables are expressed as the mean and median following interquartile ranges. A univariate analysis with the chi^2^ test was performed. P values of < 0.05 were considered statistically significant.

Statistical analyses were performed using IBM SPSS statistics version 25.0.

## Results

### Demographic data

A total of 710 consecutive operations, representing 663 patients, were included, with a female predominance (55.4%) and mean age of 65.7 (18–96) years. (Table 1).

**Table 1:** Demography. Baseline characteristics for all operations

		n (%)	Mean age
*All operations*		710 (100%)	
Gender	Female	393 (55.4)	66.2
	Male	317 (44.6)	65.0
Age	All operations	18 to 96 years	65.7
ASA-classification	ASA-1	72 (10.1)	49.1
	ASA-2	247 (34.8)	59.0
	ASA-3	282 (39.7)	71.9
	ASA-4	98 (13.8)	75.8
	ASA-5	11 (1.5)	72.5
Priority	< 30 min	25 (3.5)	53.6
	< 2-h	272 (38.3)	65,3
	< 6 h	413 (58.2)	66.7
Surgery	Primary	597 (84.1)	65.8
	Reoperation	113 (15.9)	64.9
Comorbidity	Yes (one or more)	388 (51.8)	68.1
	No	342 (48.2)	63.0
	Obesity	88 (12.4)	55.9
	Smoker	92 (13.0)	62.1
	Renal failure	90 (12.7)	77.1
	IHD	84 (11.8)	77.0
	COPD	57 (8.0)	74.3
	Heart failure	63 (8.9)	78.4
	Diabetes	82 (11.5)	72.3
	BMI n = 640	Mean 25.57	
Cancer diagnosis^a^	Yes	218 (30.7)	72.7

*ASA* American society of anaesthesiologist-classification of comorbidity. Priority, for emergency the guideline to start of surgery. Obesity classified as BMI > 30. *IHD* ischemic heart disease. *COPD* chronic obstructive pulmonary disease. *BMI* length and weight was detected in 640 cases

^a^Cancer diagnosis of any kind from 3 years before until 1 year after surgery

In 51.8% of the operations, the patient suffered from one or more of the defined comorbidities (smoking, diabetes, renal failure, heart failure, IHD, COPD and obesity). When the prevalence of the different comorbidities was explored, it was found in between 8.0 and 13.0 per cent respectively. The group with a cancer diagnosis accounts for almost a third of the cohort with a mean age of 72.7 years (Table 1).

For the cohort, bowel obstruction represented the most common underlying pathology in 56.7% of all operations, with the small intestine as the most common site. Different intestinal perforations were seen in 30.5% of the cases and peritonitis in 26.3% (Table 2).

**Table 2:** Case demography presented as pathology:

	n (%)
All operations	710 (100%)
All Ileus	403 (56.7)
Ileus-small intestine	334 (47.0)
Ileus-colon	69 (9.7)
All intestinal perforations	217 (30.5)
Perforated colon	60 (8.5)
Perforated small intestine	80 (11.3)
Gastric perforation	56 (7.9)
Anastomosis leakage	21 (3.0)
Peritonitis	187 (26.3)
Faecal peritonitis	57 (8.0)
Purulent peritonitis	43 (6.1)
Other peritonitis	87 (12.3)
Intestinal ischemia/gangrene	81 (11.4)
Intraabdominal bleeding	43 (6.1)
Trauma laparotomy	21 (3.0)

This table is a presentation of the pathology that represent the underlying need for surgery among the 710 operations

The cohort contains 41 patients who had EL more than once (1 patient had 4 operations, 4 patients had 3 operations and 36 patients had 2 operations) and these 41 patients therefore represent 88 operations. The condition for including this category of cases is that the procedure was unexpected in the sense that the patient improved clinically from the acute condition to the point of being discharged/or ready for discharge, from the hospital, but then suffered a new event that mandated an EL. The time span could be weeks, months or years after the index emergency laparotomy. In cases where this occurred, the mortality variable has been adjusted so that an expiry can only be attributed as a result to the last EL. However, variables for overall hospital stay, ICU time or other morbidity have been left unchanged. The cohort thus contains 663 unique patients.

### Perioperative management analysis

The time from notification to operate until the first incision was analysed; the 30-min group’s mean time to start was 1:01 (hours:minutes), the two-hour priority group had a mean start of 2:33 h and the mean for six-hours’ priority was 4:43. The overall mean for the entire cohort was 3:46 and, in 87% of all cases, the first incision was made within six hours (Table 3). The most common level of surgical competence in the operating theatre (OT) was a senior consultant (63.6%) with a registrar in 8.6% of all operations. For anaesthesiologists, the presence of a senior consultant in theatre was seen in 42.0% of all cases, while the figure for registrars was 38.7% (Table 4). The 30-day and one-year mortality for patients who received intestinal anastomosis were 10.1%/20.8% and for intestinal stoma 18.0%/41.6% respectively (Table 5).

**Table 3:** In hospital management—time data

	n	Mean (median, i.q.r)	Total
		*(Days:hours)*	*(Days)*
Overall hospital stay	710	12:03 (7:12, 9:01)	8617
All ICU admissions	169	5:10 (2:03, 5:19)	918
		*(Hours:minutes)*	*(Hours)*
Surgical operation time	710	1:30 (1:21, 1:00)	1065
Care at recovery ward	585	7:36 (5:50, 5:24)	4446
Priority			
< 30 min	25	1:01 (0:45, 1:08)	
< 2-h	272	2:33 (2:16, 1:25)	
< 6-h	413	4:43 (3:58, 2:42)	
All operations	710	3:46 (3:02, 2:40)	

This table is a summary of the length of stay (LOS) in hospital, LOS at ICU and LOS at recovery ward

Total column for overall hospital stays represents all hospital days for all cases in the study (8617 days), including days at the ICU, hours of surgery and in recovery ward. Care at recovery ward does not include cases admitted to ICU directly from theatre

Priority shows time from booking of the operation to first incision, related to priority. *i.q.r*  interquartile range

**Table 4:** Operation theatre management

	n (%)
Primary surgery	597 (84.1)
Reoperation	113 (15.9)
*Surgical procedure*	
All intestinal resections	252 (35.5)
Resection of small intestine	155 (21.8)
Resection of colon	84 (11.8)
Resection of small intestine and colon	14 (2.0)
Resection of other abdominal organ	15 (2.1)
Intestinal stoma	178 (25.1)
Intestinal anastomosis	159 (22.4)
Adhesiolysis	320 (45.1)
*Anaesthesiologic care*	
Induction drug propofol	593 (83.5)
Induction drug ketamine and propofol	52 (7.3)
Induction drug ketamine	62 (8.7)
Thoracic epidural anaesthesia	477 (67.2)
Treated with vasopressor infusion	505 (71.1)
Arterial-line inserted	253 (35.6)
Anaesthesiologic complication- aspiration	5 (0.7)
Perioperative treatment with antibiotics	555 (78.2)
*Level of competence in the OT*^*a*^	
Surgical registrar	61 (8.6)
Specialist in surgery	196 (27.8)
Senior consultant in surgery	450 (63.6)
Anaesthesiologic registrar	273 (38.7)
Specialist in anaesthesiology	136 (19.3)
Senior consultant in anaesthesia	296 (42.0)

This table shows the management variables examined

*OT* operation theatre

^a^ Missing value. Surgery n = 3, Anaesthesiology n = 5

**Table 5:** Outcome related to management

Mortality n (%)		Total number in the cohort	30-days	365-days	Not deceased
			Accumulated mortality n (%)		
All operations		710	101 (14.2)	189 (26.6)	521 (73.4)
Priority	< 30 min	25	6 (24.0)	7 (28.0)	18 (72.0)
	< 2-h	272	59 (21.7)	87 (32.0)	185 (68.0)
	< 6-h	413	36 (8.7)	95 (23.0)	318 (77.0)
*Surgical procedure*					
Intestinal resection	Small intestine	155	20 (12.9)	34 (21.9)	121 (78.1)
	Colon	84	16 (19.0)	29 (34.5)	55 (65.5)
	Small intestine and colon	14	6 (42.9)	7 (50.0)	7 (50.0)
Intestinal anastomosis		159	16 (10.1)	33 (20.8)	126 (79.2)
Intestinal stoma		178	32 (18.0)	74 (41.6)	104 (58.4)
Adhesiolysis		320	36 (11.3)	79 (24.7)	241 (75.3)
Resection of abdominal organ		15	4 (26.7)	8 (53.3)	7 (46.7)
*Anaesthesiologic care*^*a*^					
Induction drug propofol		592	53 (9.0)	122 (20.6)	470 (79.4)
Induction drug ketamine and propofol		52	15 (28.8)	26 (50.0)	26 (50.0)
Induction drug ketamine		62	31 (50.0)	39 (62.9)	23 (37.1)
Thoracic epidural anaesthesia		477	42 (8.8)	99 (20.8)	378(79.2)
Treated with vasopressor infusion		505	94 (18.6)	164 (32.5)	341 (67.5)
A-Line inserted		253	55 (21.7)	100 (39.5)	153 (59.5)
Anaesthesiologic complication- aspiration		5	3 (60.0)	4 (80.0)	1 (20.0)
Treatment with antibiotics		555	84 (15.1)	162 (29.2)	393 (70.8)
*Competence*					
Highest level of competence in the operation theatre^b^	Surgical registrar	61	5 (8.2)	12 (19.7)	49 (80.3)
	Specialist in surgery	196	19 (9.7)	37 (18.9)	159 (81.1)
	Senior consultant—surgery	450	76 (16.9)	138 (30.7)	312 (69.3)
	Anaesthesiologic registrar	273	30 (11.0)	72 (26.4)	201 (73.6)
	Specialist in anesthesiology	136	18 (13.2)	28 (20.6)	108 (79.4)
	Senior consultant—anaesthesia	296	50 (16.9)	86 (29.1)	210 (70.9)
*ICU*					
All ICU admissions^c^		169 (23.8)	64 (37.9)	88 (52.1)	81 (47.9)
Direct ICU admission from operation theatre		126 (17.7)	56 (44.4)	71 (56.3)	55 (43.7)

Priority, for emergency the guideline from notification to operate until first incision

^a^Missing value induction drug n = 4

^b^Missing value. Surgery n = 3, Anesthesiology n = 5

^c^All ICU admissions represents all admissions for a patient to ICU during hospital stay after emergency laparotomy

### Outcome analysis

The overall short-term mortality (30 days) following the 710 ELs in 663 patients was 14.2%, while long-term mortality (365 days) was 26.6% (Table 5). The group with ICU admission at any time during the hospital stay showed a 30-day mortality rate of 37.9% and this group (n = 169) accounts for almost a quarter (23.8%) of the cohort (Table 5). In the group that underwent multiple surgeries (41 patients and 88 operations), three patients died within 30 days of surgery; in one case there were only weeks between the procedures and the mortality for the first EL was adjusted. However, the short-term mortality in this group is low compared with the entire cohort; if calculated on all 88 operations, it is 3.4% and, if calculated on the 41 patients, it is 7.3%. Overall mortality is presented in relation to all 88 operations (Table 6).

**Table 6:** Outcome related to demography for all operations

		Total number in the cohort	30-days	365-days	Not deceased
			Accumulated mortality n (%)		
All operations		710	101 (14.2)	189 (26.6)	521 (73.4)
Primary surgery		597	85 (14.2)	149 (25.0)	448 (75.0)
Re-operation		113	16 (14.2)	39 (34.5)	74 (65.5)
Age Mean age for all operations 65,6 (years)	18–29	34	2 (5.9)	2 (5.9)	32 (94.1)
	30–39	39	0 (0.0)	1 (2.6)	38 (97.4)
	40–49	65	2 (3.1)	6 (9.2)	59 (89.8)
	50–59	92	5 (5.5)	13 (14.2)	79 (85.8)
	60–69	127	11 (8.7)	25 (19.7)	102 (80.3)
	70–79	164	29 (17.7)	56 (34.1)	108 (65.9)
	80–89	167	41 (24.6)	72 (43.1)	95 (56.9)
	90–100	22	11 (50.0)	14 (63.6)	8 (36.4)
Gender	Female	393	56 (14.2)	106 (27.0)	287 (73.0)
	Male	317	45 (14.2)	83 (26.2)	234 (73.8)
Comorbidity^a^	Yes (one or more)	368	66 (17.9)	113 (30.7)	255 (69.3)
	None of the below	342	35 (10.2)	76 (22.2)	266 (77.8)
	COPD	57	17 (29.8)	26 (45.6)	31 (54.4)
	IHD	84	25 (29.8)	37 (44.0)	47 (56.0)
	Heart failure	63	22 (34.9)	32 (50.8)	31 (49.2)
	Diabetes	82	12 (14.3)	30 (36.6)	52 (63.4)
	Renal failure	90	25 (27.8)	42 (46.7)	48 (53.3)
	Obesity	88	9 (10.2)	14 (15.9)	74 (84.1)
	Smoker	92	11 (11.9)	19 (20.7)	73 (79.7)
Cancer diagnosis^b^		218	33 (15.1)	100 (45.9)	118 (54.1)
ASA-classification	ASA 1	72	2 (2,8)	5 (6.9)	67 (93.1)
	ASA 2	247	8 (3.2)	24 (9.7)	223 (90.3)
	ASA 3	282	42 (14.9)	92 (32.6)	190 (67.4)
	ASA 4	98	44 (44.9)	61 (62.2)	37 (37.8)
	ASA 5	11	5 (45.5)	7 (63.6)	4 (36.4)
Multiple surgery^c^		88	3 (3.4)	12 (13.6)	76 (86.4)
*Pathological findings*					
Ileus	Small intestine	334	28 (8.4)	60 (18.0)	274 (82.0)
	Colon	69	9 (13.0)	24 (34.8)	45 (65.2)
Intestinal perforation	Colon	60	18 (30.0)	24 (40.0)	36 (60.0)
	Small intestine	80	19 (23.8)	29 (36.3)	51 (63.7)
	Ventricle	56	11 (19.6)	20 (35.7)	36 (64.3)
	Anastomosis leakage	21	4 (19.0)	6 (28.6)	15 (71.4)
Peritonitis	Purulent	43	7 (16.3)	12 (27.9)	31 (72.1)
	Faecal	57	14 (24.6)	25 (43.9)	32 (56.1)
	Other peritonitis	87	24 (27.6)	33 (37.9)	54 (62.1)
Intestinal ischemia/gangrene		81	21 (25.9)	30 (37.0)	51 (63.0)
Abdominal bleeding		43	10 (23.3)	14 (32.5)	29 (67.5)
Trauma laparotomy		21	2 (9.5)	4 (19.0)	17 (81.0)

*ASA* American society of anaesthesiologist-classification of comorbidity

^a^“Yes” related to comorbidity means that the patient had one or more of the specified comorbidities below. Ischemic heart disease (IHD), chronic obstructive pulmonary disease (COPD), Obesity classified as BMI > 30

^b^Cancer diagnosis of any kind from 3 years before until 1 year after surgery

^c^The cohort contains 41 patients who had EL more than once (1 patient had 4 operations, 4 patients had 3 operations and 36 patients had 2 operations), these 41 patients therefore represent 88 operations. Mortality variable has been adjusted so that an expiry only can be attributed as a result to the last of a patients operations

In the univariate analysis, mortality was strongly correlated to ASA classification, ICU care, all kinds of peritonitis, IHD, renal failure, heart failure, COPD and cancer diagnosis. Gender was not correlated to mortality (Table 7). Dividing the cohort into groups based on surgical complications (i.e. minor and major complications) revealed that the minor group (Clavien–Dindo score of 1 to 3a) represented 62.0% of the cohort (Table 8).

**Table 7:** Factors related to mortality in univariate analysis

Mortality		30 days (%)	365 days (%)	p-value
		Accumulated mortality		
Gender	Female	14.2	27.0	0.695
	Male	14.2	26.2	
ASA-classification	1	2.8	6.9	< 0.001
	2	3.2	9.7	
	3	14.9	32.6	
	4	44.9	62.2	
	5	45.5	63.6	
Pathology, peritonitis	None	10.7	22.8	
	Purulent	16.3	27.9	
	Faecal	24.6	43.9	
	Other	27.6	37.9	< 0.001
Comorbidity-any	None	10.2	22.2	
	Yes	17.9	30.7	0.020
Renal failure	None	12.3	24.4	
	Yes	27.8	46.7	< 0.001
IHD	None	12.3	25.1	< 0.001
	Yes	29.8	44.0	
COPD	None	13.0	25.9	0.008
	Yes	29.8	45.6	
Heart failure	None	12.4	25.0	< 0.001
	Yes	34.9	50.8	
Cancer diagnosis*	None	13.8	18.1	< 0.001
	Yes	15.1	45.9	
Admitted to ICU at any time	None	7.0	19.5	< 0.001
	Yes	37.9	52.1	

Univariate analysis performed using Chi2-test. *ASA* American society of anaesthesiologist-classification of comorbidity. “Comorbidity-any” represents one or more of the following: Renal failure, IHD—ischemic heart disease, COPD—Chronic obstructive pulmonary disease, heart failure, Diabetes, Obesity and smoker. *Cancer diagnosis of any kind from 3 years before until 1 year after surgery. *ICU* intensive care unit

**Table 8:** Surgical complications

Clavien–Dindo score group	Missing n (%)	1–3a n (%)	3b–5 n (%)	Total
	7 (1.0)	440 (62.0)	263 (37.0)	710

This table shows the proportion of cases that had a surgical complication according to Clavien–Dindo classification. During data collection, a high classification automatically erase a lower

## Discussion

The current study investigates the outcome (i.e. mortality, overall LOS in hospital, the need for intensive care and complications) and perioperative management (i.e. surgical care, anaesthesiological care, time to first incision and timepoint analysis) in a cohort of adult patients undergoing EL.

The overall short-term mortality after EL is 14.2%, which is comparable to recent studies in the field (range from 7 to 21%) [6, 8, 10, 13, 14, 18]. The mean overall LOS in hospital was just over 12 days, which is slightly shorter than other cohorts [8, 13]. Almost a quarter (23.8%) of the patients were admitted to the ICU at any time, with a mean ICU stay of almost five and a half days. In the Danish study by Tengberg et al., 21.8% of the patients in a similar cohort of controls were admitted to the ICU during their hospital stay [13]. The short-term mortality for the cohort admitted to the ICU was 37.9%, which is at the same level as previous studies [10, 19].

Cohort demographics are similar to several others, although the mortality in different studies varies and this cohort shows a mortality somewhere in the middle segment of those reported [6, 8, 10, 13, 18–20]. Given that the mortality after emergency laparotomy is so high, it is desirable to try to improve the outcome. The importance of working on this issue cannot be overemphasized.

The perioperative outcome analysis shows a significant association with comorbidity, critical illness, old age and a cancer diagnosis in relation to mortality. Previous studies also show a correlation between mortality and ASA classification, as well as ICU admissions [10, 13]. The data demonstrate the widely accepted fact that the elderly who carry a greater disease burden run a high risk of having a poorer outcome postoperatively [6]. This is also supported by the large difference in short-term mortality among the different age groups in the cohort. Furthermore, almost half the patients appear not to have any comorbidity. This is perhaps somewhat underestimated, as our analysis of existing comorbidity was limited to obesity, smoking, renal failure, IHD, heart failure and diabetes. As a result, several patients may be registered as “None" in the variable of comorbidity and still carry a significant disease burden (e.g. neurological and psychiatric conditions).

Data on medical competence in the OT show a clear difference in formal competence between surgeons and anaesthesiologists. The finding indicates that, to a large extent, junior anaesthesiologists manage patients undergoing major surgery and anaesthesia by themselves. However, for both surgeons and anaesthesiologists, the short-term mortality is lowest in the group of junior physicians, indicating that the critically ill patients were managed by a more senior colleague.

As expected, short-term mortality is higher in the groups where surgery was deemed more urgent (i.e. operation planned within 30 min or 2 h). This most probably represents the seriousness of the underlying condition, as well as the physical condition of the patient at the time of decision-making. Long-term mortality differs less between the priority groups, thereby indicating that other factors, such as age, underlying malignant disease and so on, are important for long-term survival. Furthermore, we can see from our data that the patients who received intestinal anastomosis have a clearly lower short and long-term mortality and that the patients who underwent procedure with intestinal stoma formation show a clearly higher mortality. This is—in our interpretation—related to the surgical strategy where the surgeons in our department are less prone to perform an intestinal anastomosis in a patient who is critically ill and with deranged organ functions. Further in-depth-analyses of mortality in relation to confounding factors for patients undergoing EL are needed.

To ensure that no operations were missed or incorrectly selected, all laparotomies were collected, even laparotomies where the primary intervention code was different (i.e. when the surgeon notified the need to operate, the primary plan was to perform another surgical procedure, for example, only a laparoscopy, but a perioperative change of plan led to a laparotomy). All medical records were scrutinised to exclude/include every specific case and this process resulted in the complete list of 710 operations. If the cohort was based exclusively on laparotomy as a surgical procedure in the registers, the cohort would most probably not reflect reality, because operations would probably have been missed or incorrectly included.

It can be argued that the 41 patients that underwent multiple surgeries, thereby representing more than one operation each, change the demographics of the cohort. We believe that unexpected new operations comprising emergency laparotomies are important to include, as the current study focuses heavily on perioperative management. On the other hand, if the unexpected new operations were excluded, the cohort would not reflect reality. However, it is important to adjust outcome variables so that data on time points, complications and mortality are accurate. The inclusion of several operations connected to one individual could skew the mortality data, especially since the study group was chosen to account for the mortality of the last performed laparotomy. It appears that the mortality rate in the group that undergoes multiple surgery is very low and has little impact on the overall mortality for the entire cohort.

This study has several limitations. It is a single-centre study, which risks giving data less external validity. The data in the study are prospectively recorded but retrospectively collected from the patients’ medical journals. The validity of data collected in this way depends on the accuracy of medical records, in which hundreds of healthcare professionals have entered information about the patients undergoing surgery. On the other hand, it can be argued that, with so many professionals involved, external validity increases. Moreover, the study period is relatively long, a little more than three years, which offers the scope for changes in treatment during the study period.

Multivariate analyses of the significant results presented here have not been performed. These analyses will be performed when the control cohort can be compared with the intervention group in the SMASH study. In spite of this, the main results presented in this study are consistent with those in previously published studies using comparable populations [8, 10, 13, 14].

In a Swedish context, this cohort study is the first major review of a large population that has undergone EL. Acute high-risk abdominal surgery accounts for a considerable proportion of acute surgical care in healthcare systems worldwide [4]. Underlying pathologies are a major contributor to mortality and morbidity in these patients, but studies indicate that standardised management can improve the outcome [8, 13, 14, 19]. It is therefore of great importance to further study this group of patients. Our results present the first part of the SMASH project [21], an ongoing study at the Northern Älvsborg County Hospital (NÄL), NU-Hospital Group (Trollhättan, Sweden) exploring whether or not the introduction of a standardised perioperative protocol for high-risk abdominal surgery can affect outcome in a Swedish context.

The EL is a lifesaving procedure and a cornerstone of acute surgical care. Relatively few studies of outcome and perioperative management are carried out and there is a need for more knowledge in the field.

## Conclusion

This study reveals mortality in the same range as previous studies. We perceive that the mortality rate is high and intend to investigate whether the outcome after EL in Sweden can be improved. It has not yet been shown whether this is possible by standardising perioperative care.

## Data Availability

The datasets analysed during the current study are available from the corresponding author on reasonable request.

## Abbreviations

EL: Emergency laparotomy
ICU: Intensive care unit
LOS: Length of stay
OT: Operating theatre
NÄL: Norra Älvsborgs County Hospital
C–D: Clavien–Dindo
